# A multi-objective optimization approach for integrated risk-based internal audit planning

**DOI:** 10.1007/s10479-023-05228-2

**Published:** 2023-02-14

**Authors:** Xiong Wang, Fernando A. F. Ferreira, Pengyu Yan

**Affiliations:** 1 ISCTE Business School, BRU-IUL, University Institute of Lisbon, Lisbon, Portugal; 2 School of Management and Economics, University of Electronic Science and Technology of China (UESTC), Chengdu, Sichuan People’s Republic of China; 3 Fogelman College of Business and Economics, University of Memphis, Memphis, TN USA

**Keywords:** Integrated audit plan, Risk assessment, Analytic hierarchy process, Fuzzy comprehensive evaluation, Weighted multi-choice goal programming

## Abstract

Annual audit planning is a multi-criteria decision-making problem faced by internal audit departments of all organizations. Due to the constrained audit resources, the planning process primarily involves the analysis and evaluation of complex factors for selecting auditable units that maximize the full potential of internal audit. Previous research on internal audit planning only focused on the goal of risk minimization and applied ranking methods to prioritize alternatives. In order to enable internal audit activities to add more value to the organization, the integrated risk-based internal audit planning is proposed to assist audit department in achieving multiple objectives in addition to risk management. Meanwhile, a multi-stage framework is proposed to support the development of such value-added internal audit plan. The new framework integrates the risk assessment of auditable units with the selection of audit activities and resource allocation through a combined analytic hierarchy process (AHP), fuzzy comprehensive evaluation (FCE) and weighted multi-choice goal programming (WMCGP) approach. The model considers both qualitative and quantitative decision criteria. A real-life case study of the development of an integrated risk-based annual audit plan is presented, and sensitivity analysis is performed to illustrate the validity of the proposed approach. The results indicate that the proposed framework is a useful tool for internal audit planning and the implications of the study can be extended to various selection and allocation problems.

## Introduction

Internal audit is regarded as an integral component of corporate governance by providing independent and objective assurance, advice, and insight to stakeholders (Behrend & Eulerich, 2019). As the first phase of internal audit cycle, internal audit planning defines the topic, scope, resources, and schedule of the audit engagement to be performed (Menekse & Camgoz-Akdag, 2022). In general, the plan is developed each year and thus the process is also called annual audit planning. As the saying goes, *“well begun is half done”*. Effective internal audit planning enables the internal audit function (IAF) to better use scarce resources, and directs internal auditors’ attention to critical and value-added areas. In contemporary organizations with a wide variety of auditable units (or auditable areas), the core processes of internal audit planning are the selection of potential audit topics and allocation of scarce audit resources (*e.g.*, audit time and budget). Annual audit planning is a complex and challenging decision-making problem. Therefore, a structured and systematic procedure with mathematical models becomes necessary to assist with the decision.

Risk-based audit planning is considered as a basic characteristic of modern internal auditing (Eulerich et al., 2020). Prior research on internal audit planning focused on prioritizing/ranking auditable areas only according to the risk level. Although a key mission of internal auditing is to mitigate major risks impacting an organization, excessive focus on risks can prevent the IAF from appropriately considering organizational strategy, and has the potential to put the IAF in an unfavorable light (Pitt, 2014). For instance, the IAF may need to conduct advisory services in areas that are not high risk but important to achieve organizational objectives (IIA, 2020). Aditya et al. (2018) and Kotb et al. (2020) also pointed out that the audit plan should be agile, insightful, relevant, forward-looking, risk-oriented, and aligned with business strategy. To this end, we propose an integrated risk-based approach to internal audit planning, which differs from the conventional risk-based approach in the coverage of value-added factors and goals. In other words, not only the risk management but also other audit missions will be taken into consideration when determining if the audit activities should be carried out. The integrated risk-based internal audit planning provides a more holistic view to effectively allocate resources to areas where auditors can have the most impact, increasing the relevance and credibility of internal audit activities, and delivering increased value to the organization.

The integrated risk-based audit planning is a multi-stage, multi-criteria, and multi-objective decision-making practice. The process involves risk assessment, auditable area selection as well as resource allocation, which requires strong analytical skills. Notwithstanding, more than half of the respondents in a global survey indicated that the usage of technology tools in risk assessment or audit planning was “none” or “minimal” (Cangemi, 2015). As an analytical approach of problem-solving and decision-making, operational research (OR) techniques can be applied to a variety of real-world use cases. Motivated by the significance of the internal audit planning, the advantages of integrated risk-based approach, the performance gaps, and the gaps between practice and extant literature pointed out by Roussy and Perron (2018), the purpose of this paper is to present a systematic scheme with algorithmic solutions for internal audit planning problem using OR methods. Specifically, in terms of risk assessment, analytic hierarchy process (AHP) is adopted to estimate the weights of risk items, and then fuzzy comprehensive evaluation (FCE) is utilized to obtain the overall risk of each auditable area. Based on the risk assessment results and multiple objectives, the areas to be audited and the allocated audit time can be determined simultaneously using weighted multi-choice goal programming (WMCGP). To the best of authors’ knowledge, these techniques have not been used for audit planning problem.

The reasons for the use of a combined AHP-FCE-WMCGP approach are elaborated as follows.

- The AHP method (Saaty, 1980) is the most widely used multiple criteria decision analysis (MCDA) method in practice to calculate criteria weights (Vinogradova-Zinkevič et al., 2021). Although it is subject to some criticisms, it is a powerful yet simple analysis tool. For example, one major concern on AHP is the low consistency of pairwise comparisons (Rezaei, 2015). However, checking the consistency of human judgements is one of the AHP steps. In case that the judgement matrix does not meet consistency ratio threshold, remedies can be made by reperforming the pairwise comparisons, excluding the inconsistent matrix, or repairing inconsistent matrix using particle swarm optimization (PSO) technique (Bandichode et al., 2018). During the risk assessment of audit planning, pairwise comparison is also not cumbersome due to the limited evaluation criteria. Moreover, it is easy for practitioners to use available AHP software that is mature and user friendly. The AHP can be used for group decision making by aggregating group opinions. The most commonly used methods are the arithmetic mean and the geometric mean, which are considered to be aggregation by means of direct information (Coffey & Claudio, 2021). Forman and Peniwati (1998) proposed that geometric mean aggregation should be utilized when individuals pool judgements in a way that the group functions as a new individual decision-maker. Accordingly, when applying AHP in the context of group decision, this study adopts the geometric mean method to aggregate individual judgements since the group is assumed to act together as a unit.
- As an application of the fuzzy set theory (Zadeh, 1965), the FCE method is well suited for assessing the overall risk level of an auditable unit, which is a complicated, vague, and multi-level process. In performing risk assessment, practitioners usually prefer to use linguistic terms such as low, moderate, high, and significant (Ameyaw & Chan, 2015). However, previous studies on risk assessment of internal audit planning rarely considered the vagueness (Menekse & Camgoz-Akdag, 2022). Embracing the weighting vector of the evaluation factor obtained from AHP, FCE provides an approach to model and quantify ambiguous and subjective assessment judgments in the context of group decision. FCE model has been adopted in numerous assessment processes in uncertain situations. It is easy to be understood and used by DMs who are not experts in the OR field.
- Compared with other programming models, multi-choice goal programming (MCGP) (Chang, 2007) improves traditional goal programming (GP) model by considering multi-aspiration levels (*e.g.*, the more the better for benefit goal, and the less the better for cost goal) when solving multi-objective problems, and thus avoids underestimation of the decision and obtains solutions with minimum aggregate deviation/maximum aggregate achievement for all multiple goals. The WMCGP model proposed by Ho (2019) further improves MCGP, allowing DMs to emphasize objectives which they consider more important. To achieve multiple goals for the integrated risk-based audit planning, WMCGP provides a rigorous and transparent way to select auditable units and determine the optimal level of audit effort.

The major contributions of this study are stated below.

- This is the first study presenting an integrated risk-based internal audit planning, and utilizing combined OR techniques and a new risk universe of manufacturing industry for this purpose.
- It expands the knowledge base in internal auditing and promotes interdisciplinary study. Previous research on internal auditing was predominated by testing statistical hypothesis (Kotb et al., 2020) and each phase of the internal audit cycle should be investigated in more detail (Christ et al., 2021; Roussy & Perron, 2018). By illustrating the implementation of OR techniques through a real-life case, this paper not only contributes to a better understanding of the internal audit planning process, but also sheds light on the new directions in auditing research to enhance the practical relevance.
- This study provides a reference for practitioners, internal audit association, and audit software companies. With the proposed framework, the IAF can apply simple quantitative methods to develop a value-added audit plan according to departmental strategy. Audit association (*e.g.*, the Institute of Internal Auditors) can also benefit from this study to provide guideline on risk management and audit plan development to inform better practice. In addition, internal audit software companies can embed the model into their products to improve the function.

The rest of the paper is organized as follows: Sect. 2 conducts a review of prior research on methods applied to internal audit planning process and on risk identification of manufacturing sector. Section 3 presents the proposed framework for developing an integrated risk-based audit plan. Section 4 applies the proposed framework to a real-life situation. The results and management feedback are discussed as well. Finally, conclusions and avenues for future research are discussed in Sect. 5.

## Literature review

### Internal audit planning

While there is a growing interest in the decision-making problem of internal audit planning, the body of the literature is still small. Sueyoshi et al. (2009) proposed a hybrid AHP and data envelopment analysis (DEA) model to determine the stores of a rental car company that should be audited with more urgency. AHP was applied to compute the subjective risk exposure, and DEA was adopted to obtain the objective efficiency score. Then the sum of exposure (AHP score) and operational inefficiency (1 − DEA score) was used to rank each store. However, this method cannot be generalized to auditable units without measurement criteria in common. For example, not all the processes are comparable with respect to their performance and this kind of audit topics cannot be ranked with the proposed model. Goman and Koch (2019) developed a new composite index (CI) based on the geometric mean to aggregate an overall risk score of each possible audit topic. To develop a risk-based annual audit plan, an illustrative example was given to rank 13 auditable areas. In fact, risk items can be structured in multiple levels but there was a lack of analysis of risks under the main criteria in their study. Menekse and Camgoz-Akdag (2022) proposed spherical fuzzy elimination and choice expressing reality (ELECTRE) model to support internal audit planning. The introduced method was applied to evaluate risk levels of four schools of a university and the riskiest unit should be selected for an audit. Nevertheless, risk assessment was performed in terms of the five elements of COSO internal control framework without identifying specific risk items. The method was also subject to the rank-reversal problem, which could result in obtaining incorrect results.

On the other hand, some research address resource allocation problem in addition to the prioritization of auditable areas. To minimize the total risk of an organization, Serfontein and Krüger (2016) combined loss function, AHP and method of Lagrange multipliers to aid in allocating audit resources to five internal audit projects of a gold mining company. In their study, AHP was used to determine overall risk scores of auditable areas rather than a weight estimation tool. Similarly, to support the internal audit planning of Ministry of Energy and Mineral Resources of Indonesia, Purwanto et al. (2017) also used AHP to calculate risk scores of 27 auditable units. Total working days of each auditable unit were calculated by multiplying the number of auditors with the number of working days, which were defined based on the AHP results and the audit type, respectively. An implicit assumption was made that manpower needed was in proportion to the risk level, whereas it is highly likely that auditing a high-risk area is easy and thus requires fewer headcounts, making it an invalid assumption in many situations. Wang et al. (2021) proposed fuzzy AHP and MCGP for selecting audit activities out of 28 candidate audit activities, and allocating staff time synchronously to achieve goal risk level. As the risk assessment in their study was performed at organization level, it appeared that there was no connection between the current risk level and the risk reduction in terms of each auditable unit. Moreover, a marginal effect was ignored between the risk reduction value and the allocated audit time.

### Risk universe

A prerequisite of risk assessment is to identify possible risks. Nevertheless, prior studies mostly evaluated the risk of auditable units based on risk factors without risk identification. A risk factor (*e.g.*, organizational size, degree of change, or operations complexity) is a characteristic, condition, or variable that increases the possibility of the risk. Identifying risk is necessary to ensure an accurate evaluation result.

The risk universe is a list of potential risks the company faces or might face. Although risk areas vary among organizations and industries, there are four main risks for a business (Deloitte, 2013): (1) strategic risk: business decisions or events that prevent an organization from achieving its objectives; (2) financial risk: risk associated with potential financial loss to the organization; (3) operational risk: the failure of processes, systems or events that disrupts daily business operations; and (4) compliance risk: potential exposure resulting from the violation of laws, regulations, and other standards. Each of these main risk categories can be decomposed into several secondary risks.

Manufacturing plays a critical role in both advanced economies and emerging market and developing economies (EMDEs) and generates more economic activities than other sectors (Bryson et al., 2015). Based on the literature of risk management, and the real-world risk universe shared by 7 international manufacturing companies, a generic risk universe of manufacturing industry is created and displayed as Table 1. It can be used as a starting point by any organizations for developing a unique risk universe that fits them.

**Table 1:** A generic risk universe for manufacturing industry

Main risk	Secondary risk	Risk description	Examples	References
Strategic risk (U_1_)	Governance (u_11_)	Control environment Health and safety culture Communication	Lack of control environment may lead to integrity and ethical issues and inadequate oversight processes	Birkel et al. (2019), ElKelish (2018), Portman (2013), Gartner (2020)
	Key relationship management (u_12_)	Strategic supplier Joint-venture management	Inability to secure & effectively manage relationship with strategic affiliates	Gartner (2020), Gutterman (2020), Leopizzi et al. (2020)
	Major initiatives (u_13_)	Planning and execution Measurement and monitoring Mergers and acquisitions	Ineffective planning and execution of major initiatives may result in outcomes that do not meet customer expectations and/or impact business operations	Chang and Cho (2017), Portman (2013), Gartner (2020), Gutterman (2020)
	Market dynamics (u_14_)	Competition Macro-economic factors Socio-political	Competitors' new products and services or new entrants to the market impair competitive advantage and/or growth potential	Birkel et al. (2019), Gartner (2020), Ignat et al. (2020), Leopizzi et al. (2020)
	Planning and resource allocation (u_15_)	Organization structure Strategic planning Annual budgeting/forecasting	Strategic planning process is not effective, resulting in irrelevant information and non-viable or mis-aligned goals & objectives	Birkel et al. (2019), Portman (2013), Gartner (2020), Ignat et al. (2020)
	Reputation, brand and communication (u_16_)	Community program Crisis communication Sustainability Employee satisfaction	Crisis management plan does not exist or is inadequate/out-of-date resulting in a slow response and ineffective communication to stakeholders, negatively impacting the company's reputation	Portman (2013), Gartner (2020), Gutterman (2020), Ignat et al. (2020), Leopizzi et al. (2020)
Financial risk (U_2_)	Accounting and reporting (u_21_)	Accounting Financial reporting Master data	Accounting transactions are not in accordance with GAAP/regulations, inappropriate, not reconciled and/or unsupported resulting in financial misstatements	Portman (2013), Gartner (2020), Ignat et al. (2020), Scarlat et al. (2012)
	Treasury (u_22_)	Liquidity management Capital structure Interest rate	Exposure to unfavorable volatility for foreign exchange movements may lead to significant financial loss	Portman (2013), Gartner (2020), Gutterman (2020), Scarlat et al. (2012)
	Tax (u_23_)	Income tax return Transfer pricing Value added tax	Income tax returns are not accurately reconciled to the provision and/or posted to the general ledger resulting in misstatement of tax obligations	Portman (2013), Gartner (2020), Scarlat et al. (2012), Stoel et al. (2017)
Operational risk (U_3_)	Sales & marketing (u_31_)	Product innovation Price management Customer satisfaction Product availability/quality	Inability to produce a product that meets the specifications of the customer; poor product quality may lead to customer dissatisfaction, litigation exposure and decreased sales	Portman (2013), Gartner (2020), Gutterman (2020), Leopizzi et al. (2020), Scarlat et al. (2012)
	Purchasing and supply chain (u_32_)	Supplier quality Vendor selection Logistics Inventory planning	Reliance on a sole or single source of supply is not monitored/remediated, documented, and appropriately approved	Portman (2013), Gartner (2020), Gutterman (2020), Leopizzi et al. (2020), Scarlat et al. (2012)
	Human resources (u_33_)	Recruiting Succession planning Compensation and benefits	Failure to identify and manage key personnel may result in unplanned loss of key knowledge or skills	Portman (2013), Gartner (2020), Gutterman (2020), Leopizzi et al. (2020)
	Information technology (u_34_)	Information security IT operations System development Change management	IT personnel or vendors utilize their knowledge and access to programs and data including Administrative Access privileges for fraudulent activities	Birkel et al. (2019), Portman (2013), Gartner (2020), Leopizzi et al. (2020), Subriadi and Najwa (2020)
	Physical assets (u_35_)	Property and equipment Capital project management	Inappropriate management of capital projects may result in project delay and budget overruns	Portman (2013), Gartner (2020)
	Manufacturing (u_36_)	Environment, health and safety Production reliability Business continuity planning	Inadequate environmental, health and safety programs may lead to regulatory violations, litigation exposure, financial loss and potential harm to individuals	Gartner (2020), Ignat et al. (2020), Scarlat et al. (2012), Sun et al. (2020)
Compliance risk (U_4_)	Legal (u_41_)	Records management Patents protection Law suit	The company’s current and discontinued operations and current expansion projects could have litigation exposure and reputational damage	Birkel et al. (2019), Portman (2013), Gartner (2020), Leopizzi et al. (2020)
	Regulatory (u_42_)	Anti-trust/anti-corruption Trade compliance Data protection and privacy	Failure to follow anti-trust rules and regulations may lead to significant fines, criminal violations, and civil claims	Portman (2013), Gartner (2020), Leopizzi et al. (2020)
	Standards of business conduct (u_43_)	Ethics Fraud Company policy	Company values may be harmed by unethical behaviors of employees	Portman (2013), Gartner (2020), Ignat et al. (2020), Leopizzi et al. (2020)

## A multi-stage audit planning framework

**Fig. 1:**
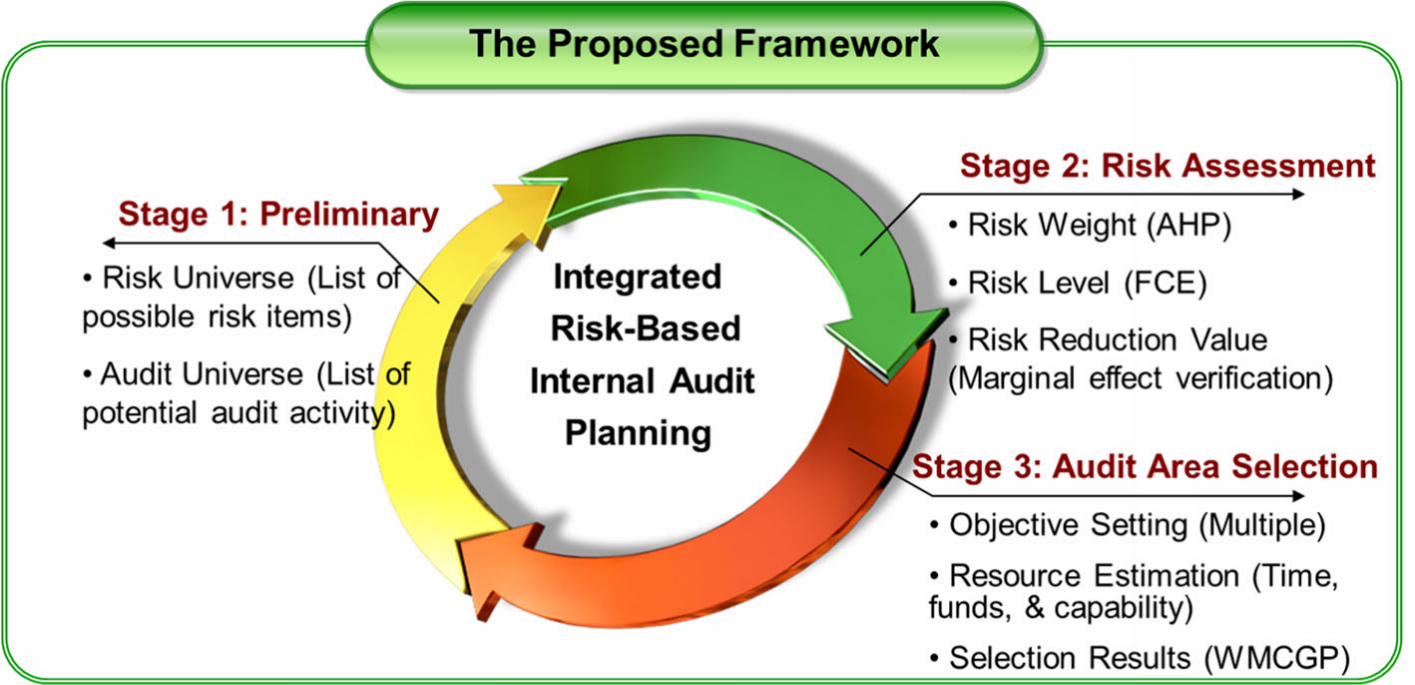
A multi-stage framework for integrated risk-based internal audit planning

To assist the IAF in making scientific and transparent decisions in the annual planning process, a multi-stage audit planning framework is proposed as Fig. 1.

As illustrated, the integrated risk-based internal audit planning is a comprehensive process that evaluates the risk levels of candidate audit areas based on the identified risk types, and then selects the areas to be audited, and determines optimal resource allocation to achieve multiple value-added goals. In the preliminary stage, possible risks in the organization are identified, and all the auditable units are listed. In the second stage, based on DMs’ judgements on the relative importance of various risks and risk rating of auditable units by risk type, the risk weights and overall risk level of each auditable unit can be computed using the AHP method and FCE method, respectively. To measure the contribution of audit activities to mitigating existing risks of auditable units, a risk reduction value is estimated. The audit can be conducted at different degrees of work scope, which requires different audit hours. The more audit effort is devoted, the more risk is reduced. However, the time spent on auditing the entity should have a decreasing marginal effect on risk reduction. In the third stage, multiple objectives are defined for the audit area selection problem. Also, available audit resources (*e.g.*, time, funds, and auditor capability) are reviewed. According to the pre-defined objectives and constraints, the audit activities to be conducted in the year and corresponding audit resources are determined using WMCGP model.

In the following sections, each stage of the proposed framework is described in detail.

### Stage 1: preliminary

To prepare a feasible audit plan, an initial effort should be made to identify organizational risks and potential areas that can be audited. The purpose of such preparation work is to define the evaluation/selection criteria and objects. Risks are potential threats that affect the achievement of organizational objectives (Jovanović et al., 2020). To identify all the key risks, a risk universe can be created by obtaining inputs through meetings, surveys, interviews and workshops with business leaders, and internal auditors’ independent research. The audit universe, a list of all the auditable units, simplifies the risk evaluation of the whole organization. The auditable units can be subsidiaries of the organization, business processes, organizational functions, or a mix of them. Gartner (2018)’s survey on 88 companies indicated that most organizations defined audit universes based on business units (73%) and processes (72%), followed by risk type (42%), geographic area (31%) and others (7%). The survey also revealed that about half of audit universes (47%) consist of 50 to 250 entities, while 30% organizations have more than 250 auditable units, and the rest 23% have fewer than 50 units.

Griffiths (2020) introduced detailed steps to establish the risk universe and audit universe from scratch in practice. A noteworthy tip is that both universes should be updated periodically to reflect the changes of internal and external environments.

### Stage 2: risk assessment

This paper adopts a specific-risk approach for risk assessment, which connects risk types and auditable units using a matrix with each auditable unit in a row and each risk in a column (Heldifanny & Tobing, 2019). When rating the risk level, evaluators are more likely to use qualitative terms (*e.g.*, low, medium, and high) than quantitative ranges or point estimates (Stoel et al., 2017). Table 2 shows the risk scale revised from Joshi and Singh (2017).

**Table 2:** Risk scale of auditable unit

Scale	Description
Significant	Risk is totally intolerable and thus requires prompt action to address the risk
High	Risk is unacceptable and should implement action plan as early as possible
Medium	Risk may be acceptable within a short period, but action is needed to reduce risk
Low	Risk is acceptable and the event does not constitute a concern. There are opportunities for further improvement, and risk mitigation should be implemented in future
Very low	Risk is slight and negligible

To prioritize various risk types according to the importance to organizational performance, an AHP questionnaire is designed to collect the data. In the meantime, a FCE questionnaire is developed to collect DMs’ risk rating data. The obtained data can be processed by commercial software or spreadsheet. Based on the calculated risk level, the risk reduction value is obtained via subjectively assessed formulas. The used methods are introduced as follows.

#### Analytical hierarchy process

The first step in using AHP is to define the criteria (*e.g.,* risk items for risk assessment problem). The key feature of AHP technique is the pairwise comparison for scoring each criterion and sub-criterion. In the pairwise comparison, any two factors are compared with each other. The comparison is usually scored according to Saaty (1980)’s nine-point scale. To ensure the reliability of subjective judgments made by DMs, consistency check should be conducted. If the consistency ratio is below 0.1, the judgement matrix is satisfactory. Otherwise, initial values of judgement matrix elements should be revised to improve the consistency. To generate the weighting vector $W = \left\{ {w_{1} ,w_{2} , \cdots ,w_{I} } \right\}$, the most widely used method is the characteristic root method, and $\sum\nolimits_{i = 1}^{I} {w_{i} }$ = 1.

#### Fuzzy comprehensive evaluation

The procedure of FCE method can be described as following steps (Hsiao & Ko, 2013).

*Step 1*: Determine evaluation factor set $U = \left\{ {u_{1} ,u_{2} , \cdots ,u_{I} } \right\}$ (*e.g.*, risk items in Table 1) and judgement set $V = \left\{ {v_{1} ,v_{2} , \cdots ,v_{J} } \right\}$ (*e.g.*, risk scale in Table 2).

*Step 2*: Setup of the fuzzy evaluation matrix. A $U$-$V$ fuzzy relationship matrix $R$ can be generated for each alternative/evaluation object (*e.g.*, auditable unit) as follows.

$$ R = {(}r_{ij} {)}_{I \times J} = \left[ {\begin{array}{*{20}c} {r_{11} } & {r_{12} } & \ldots & {r_{1J} } \\ {r_{21} } & {r_{22} } & \ldots & {r_{2J} } \\ \vdots & \vdots & \vdots & \vdots \\ {r_{I1} } & {r_{I2} } & \ldots & {r_{IJ} } \\ \end{array} } \right] $$

The process is also called fuzzy transformation. The membership function denotes the fuzziness of the evaluation factor by assigning each evaluation factor a grade of membership ranging from 0 to 1. In the context of group evaluation, $r_{ij}$ (*i* = 1, 2, ⋯, *I*; *j* = 1, 2, ⋯, *J*) is the membership degree which represents the percentage of evaluators who rated *j* th grade for *i* th factor. In other words, $r_{ij}$ = $x_{ij}$/*C* in which $x_{ij}$ means the number of evaluators who rate evaluation object as $v_{j}$ in regard to criterion $u_{i}$, and *C* is the total number of evaluators. Additionally, $\sum\nolimits_{j = 1}^{J} {r_{ij} }$ = 1.

*Step 3*: Conduct fuzzy comprehensive evaluation of the alternative. By applying the fuzzy composite operation between the weighting vector $W$ from AHP and the fuzzy relationship matrix $R$, the comprehensive evaluation result of the alternative is obtained via Eq. (1).

1 $$ D = W \circ R = [d_{1} ,d_{2} , \cdots ,d_{J} ] $$

where $\circ$ denotes the composition operator. This study uses weighted average principle and $d_{j} =\sum\nolimits_{i = 1}^{I} {(w_{i} \times } \;r_{ij} )$. To make it more straightforward for decision making, the fuzzy output is then converted into a crisp number through defuzzification process (Chen et al., 2015).

#### Risk reduction value

Prior research is quite limited to explore the relation between devoted audit time and risk reduction effect, and it is a difficult task to measure the exact relation due to complex scenarios and lots of uncertainty in practice (Hamid, 2012).

As risk cannot be fully eliminated, there is a maximum amount of risk reduction by conducting audit activities. Inspired by Miltz et al. (1991), audit work scope and corresponding risk reduction percentage ($RP_{n}$) are classified into the following four categories.

- Small scope review. Internal auditors perform interview, walkthrough, and high-level review of the auditable unit. Detailed investigation or testing is not conducted. In this scenario, the risk reduction is judgmentally set as 40% of the maximum risk reduction amount.
- Moderate scope review. Audit testing can cover all the key processes and risk reduction is set as 60% of the maximum risk reduction amount.
- Large scope review. Internal auditors perform deep testing on majority of the applicable processes. The risk reduction equals to 80% of the maximum risk reduction amount.
- Full scope review. Auditors conduct a complete and extensive review of the auditable units to achieve maximum risk reduction amount. Assume that 90% of existing risk can be mitigated at the most by conducting a full audit.

In addition, there is a decreasing marginal risk reduction to additional audit scope/effort. In other words, for each auditable unit, the broader the audit scope is, the more the risk reduction can be achieved. However, risk reduction per unit of time decreases. Audit time at different work degrees of an auditable unit can be obtained from internal auditors based on their professional judgements (*e.g.*, complexity and nature of each audit) and/or historical data (*e.g.*, timesheet which records actual audit time spent on comparable audits).

In the below, Eq. (2) expresses the above estimation and Eq. (3) examines the diminishing marginal returns of audit time.

2 $$ RR_{mn} = RP_{n} \times 0.9Z_{m} $$

3 $$ \frac{{\Delta RR_{mn} }}{{\Delta T_{mn} }} > \frac{{\Delta RR_{m(n + 1)} }}{{\Delta T_{m(n + 1)} }} $$

where $RR_{mn}$ indicates risk reduction value of $m$ th auditable unit at $n$ th audit scope; $RP_{n}$ means risk reduction percentage under $n$ th work scope, thereby $RP_{1}$, $RP_{2}$, $RP_{3}$, and $RP_{4}$ equal to 40%, 60%, 80%, and 100%, respectively; $Z_{m}$ denotes pre-audit risk score of $m$ th auditable unit as per FCE results; $T_{mn}$ means audit hours spent on $m$ th auditable unit at $n$ th audit scope. $\Delta T_{mn}$ (or $\Delta T_{{m\left( {n + 1} \right)}}$) indicates additional time to conduct audit in $m$ th auditable unit at one level higher than $n$ (or $n$ + 1); $\Delta RR_{mn}$ (or $\Delta RR_{m(n + 1)}$) indicates additional risk reduction achieved by expanding audit scope at one level higher than $n$ (or $n$ + 1) when working on $m$ th auditable unit.

### Stage 3: audit area selection

As discussed earlier, a value-added audit plan is not only risk-focused but also integrated, proactive and future-focused, such as incorporating organizational strategy and business needs. Meanwhile, scarce audit resources are constraints for the IAF to achieve desired goals.

The selection of auditable units and determination of the audit scope can be solved simultaneously. When no audit scope is assigned to the candidate audit areas, the audit activities will not be included in the annual audit plan. The proposed WMCGP method is used to determine the audit activities with corresponding audit scope. A summary of WMCGP model is presented as Eq. (4) to Eq. (10) (Ho, 2019).

4 $$ {\text{Min}}\,\,\sum\limits_{k = 1}^{K} {w_{k} (\alpha_{k} d_{k}^{ + } + \beta_{k} d_{k}^{ - } + e_{k}^{ + } + e_{k}^{ - } )} $$

5 $$ \begin{gathered} {\text{s}}.{\text{t}}. \hfill \\ f_{k} (x) - d_{k}^{ + } + d_{k}^{ - } = y_{k} ,\,\,\,\,\,k = 1,2,...,K, \hfill \\ \end{gathered} $$

6 $$ y_{k} - e_{k}^{ + } + e_{k}^{ - } = g_{k,\max } ,\,\,\,\,k = 1,2,...,K,\,\,\,\,\left( {\text{for the case of the more the better}} \right) $$

7 $$ y_{k} - e_{k}^{ + } + e_{k}^{ - } = g_{k,\min } ,\,\,\,\,k = 1,2,...,K,\,\,\,\,\left( {\text{for the case of the less the better}} \right) $$

8 $$ {\kern 1pt} g_{k,\min } \le y_{k} \le g_{k,\max } ,\,\,\,\,k = 1,2,...,K, $$

9 $$ d_{k}^{ + } ,{\kern 1pt} {\kern 1pt} {\kern 1pt} d_{k}^{ - } ,{\kern 1pt} {\kern 1pt} e_{k}^{ + } ,{\kern 1pt} e_{k}^{ - } \ge 0,\,\,\,\,k = 1,2,...,K, $$

10 $$ {\kern 1pt} x \in F,\,\,\,\,(F{\text{is a feasible set}},\,x\,{\text{is unrestricted in sign}}) $$

where Eq. (4) is the objective function to minimize the aggregate deviation from all goals. Equation (5) to Eq. (8) determine the rang of aspirational levels and drive the target value to get closer to the upper (lower) bound.$w_{k}$ represents the weight of $k$ th goal, and $\sum\nolimits_{k = 1}^{K} {w_{k} }$ = 1; $\alpha_{k}$ and $\beta_{k}$ respectively denote the penalty weights attached to deviations $d_{k}^{ + }$ and $d_{k}^{ - }$, which are overachievement and underachievement of $k$ th goal. $e_{k}^{ + }$ and $e_{k}^{ - }$ are positive and negative deviations between aspiration value of $k$ th goal ($y_{k}$) and lower/upper bound of corresponding aspiration value ($g_{k,\min }$ or $g_{k,\max }$). ${\kern 1pt} f_{k} (x)$ is the objective function of $k$ th goal. $y_{k}$ is a continuous variable with a range of interval values.

## A real life case

A real-life case is presented to illustrate how the proposed multi-stage framework can be applied to develop an integrated risk-based audit plan. The case study is conducted at an automobile parts manufacturing company. With its global headquarter located in China, the company has 19 plants, 8 sales offices, 8 technical support centers, 5 research and development centers, and 4 logistics centers across the world. Currently there are 10 members in the IAF, who are all based at corporate headquarter. In addition, according to the secondment agreement with a multinational consulting firm, the in-house internal audit team could hire external consultants in various locations to carry out some audit activities if necessary (*e.g.*, international travel restriction due to the pandemic, or insufficient specialized skills and capabilities in certain fields). Internal audit co-sourcing model allows the IAF to operate in a more flexible and cost-effective way.

In this study, we discussed the internal audit planning problem by interviewing chief audit executive (CAE) and senior audit manager. The current annual planning process mainly relies on intuitive decision and manual work as elaborated below. (1) The audit team collects potential audit areas for the following year via various inputs, such as continuous audits conducted every year, interview with business management, audit topics proposed by internal auditors, and follow-up audits of previous findings due to the severity. (2) Based on professional judgement, senior audit manager performs risk assessment by simply rating each potential audit area as “low”, “medium” or “high”. To determine whether the topic should be included in the annual audit plan, senior audit manager judgmentally classifies the potential audit activities as “yes”, “maybe”, or “no” considering the risk category and other factors, such as audit areas requested by management frequently. (3) Senior audit manager estimates hours including co-source hours needed to audit each selected area (*i.e.*, topics marked as “yes”). When available hours are less than the total hours of the proposed activities, some audit topics need to be excluded. Otherwise, potential audit topics categorized as “maybe” will be judgmentally added to the audit plan until available hours are used up.

### Implementation of the proposed framework

Based on the 45 auditable units identified as potential audit topics for the year 2022, the implementation of the proposed framework is described below.

#### Risk assessment on auditable units

Under the specific-risk approach, risk items in Table 1 are used as the evaluation criteria of auditable units. Five experts from the IAF, including three (senior) audit managers and two senior auditors, made comparison judgments on these risk items. Questionnaires were sent out for each respondent to give their own opinion for each judgment, and when the questionnaires were sent back the judgments were combined using the geometric mean and then entered as a single judgment into a model in Super Decisions V3.2 (Mu & Pereyra-Rojas, 2018), a simple easy-to-use AHP software developed by the team of the creator of the AHP method. In the group decision using geometric mean method, it is assumed that all group members have equal importance as they are all qualified professionals. As a result, the weights of risk items are obtained as Table 3. It can be concluded that operational risk (U_3_) is rated as the most important main risk to the organization. In terms of the secondary risks, the top five risk items are accounting and reporting (u_21_), sales and marketing (u_31_), governance (u_11_), regulatory (u_42_), and manufacturing (u_36_).

**Table 3:** Risk weights (Overall matrix *CR* = 0.0053)

Main risk	Area weight	Secondary risk	Item weight	Combined weight	Rank
U_1_	0.2716	u_11_	0.3534	0.0960	3
		u_12_	0.2761	0.0750	7
		u_13_	0.1097	0.0298	14
		u_14_	0.0593	0.0161	18
		u_15_	0.0922	0.0250	16
		u_16_	0.1093	0.0297	15
U_2_	0.2256	u_21_	0.5156	0.1163	1
		u_22_	0.1655	0.0373	10
		u_23_	0.3189	0.0719	8
U_3_	0.3509	u_31_	0.3274	0.1149	2
		u_32_	0.0902	0.0317	11
		u_33_	0.0858	0.0301	13
		u_34_	0.2141	0.0751	6
		u_35_	0.0504	0.0177	17
		u_36_	0.2321	0.0815	5
U_4_	0.1519	u_41_	0.2066	0.0314	12
		u_42_	0.5402	0.0821	4
		u_43_	0.2532	0.0385	9

Meanwhile, FCE method is adopted to calculate risk score. Five experts rated risk level of each auditable unit by risk item. An example of risk rating of the first auditable unit (AU_1_) is given in Table 4. As shown, three DMs voted “high” for both governance risk (u_11_) and purchasing and supply chain risk (u_32_), while the other two DMs believed that these two risks were “significant”.

**Table 4:** Experts’ judgments of risk level

Main risk	Secondary risk	Number of DMs in each risk grading					Total
		Very low	Low	Medium	High	Significant	
U_1_	u_11_	0	0	0	3	2	5
	u_12_	0	0	4	1	0	5
	u_13_	0	0	2	3	0	5
	u_14_	3	2	0	0	0	5
	u_15_	0	0	1	3	1	5
	u_16_	0	0	1	3	1	5
U_2_	u_21_	0	0	0	2	3	5
	u_22_	0	1	0	4	0	5
	u_23_	0	3	2	0	0	5
U_3_	u_31_	0	1	2	2	0	5
	u_32_	0	0	0	3	2	5
	u_33_	0	0	0	1	4	5
	u_34_	0	1	2	2	0	5
	u_35_	0	0	4	1	0	5
	u_36_	0	0	2	1	2	5
U_4_	u_41_	0	0	3	2	0	5
	u_42_	0	0	0	1	4	5
	u_43_	0	0	0	2	3	5

By normalizing the data in Table 4, the fuzzy relationship matrix can be obtained. Combing the weighting vector from Table 3, the FCE result is presented as Eq. (11).

11 $$ D_{1} = \left[ {\begin{array}{*{20}c} {0.0960} \\ {0.0750} \\ {0.0298} \\ {0.0161} \\ {0.0250} \\ {0.0297} \\ {0.1163} \\ {0.0373} \\ {0.0719} \\ {0.1149} \\ {0.0317} \\ {0.0301} \\ {0.0751} \\ {0.0177} \\ {0.0815} \\ {0.0314} \\ {0.0821} \\ {0.0385} \\ \end{array} } \right]^{{\text{T}}} \circ \left[ {\begin{array}{*{20}r} \hfill 0 & \hfill 0 & \hfill 0 & \hfill {0.6} & \hfill {0.4} \\ \hfill 0 & \hfill 0 & \hfill {0.8} & \hfill {0.2} & \hfill 0 \\ \hfill 0 & \hfill 0 & \hfill {0.4} & \hfill {0.6} & \hfill 0 \\ \hfill {0.6} & \hfill {0.4} & \hfill 0 & \hfill 0 & \hfill 0 \\ \hfill 0 & \hfill 0 & \hfill {0.2} & \hfill {0.6} & \hfill {0.2} \\ \hfill 0 & \hfill 0 & \hfill {0.2} & \hfill {0.6} & \hfill {0.2} \\ \hfill 0 & \hfill 0 & \hfill 0 & \hfill {0.4} & \hfill {0.6} \\ \hfill 0 & \hfill {0.2} & \hfill 0 & \hfill {0.8} & \hfill 0 \\ \hfill 0 & \hfill {0.6} & \hfill {0.4} & \hfill 0 & \hfill 0 \\ \hfill 0 & \hfill {0.2} & \hfill {0.4} & \hfill {0.4} & \hfill 0 \\ \hfill 0 & \hfill 0 & \hfill 0 & \hfill {0.6} & \hfill {0.4} \\ \hfill 0 & \hfill 0 & \hfill 0 & \hfill {0.2} & \hfill {0.8} \\ \hfill 0 & \hfill {0.2} & \hfill {0.4} & \hfill {0.4} & \hfill 0 \\ \hfill 0 & \hfill 0 & \hfill {0.8} & \hfill {0.2} & \hfill 0 \\ \hfill 0 & \hfill 0 & \hfill {0.4} & \hfill {0.2} & \hfill {0.4} \\ \hfill 0 & \hfill 0 & \hfill {0.6} & \hfill {0.4} & \hfill 0 \\ \hfill 0 & \hfill 0 & \hfill 0 & \hfill {0.2} & \hfill {0.8} \\ \hfill 0 & \hfill 0 & \hfill 0 & \hfill {0.4} & \hfill {0.6} \\ \end{array} } \right] = \left[ {\begin{array}{*{20}c} {0.0097} \\ {0.0951} \\ {0.2532} \\ {0.3649} \\ {0.2772} \\ \end{array} } \right]^{{\text{T}}} $$

To interpret the results, in terms of the overall risk level of the assessed auditable unit, the probability to be “very low”, “low”, “medium”, “high”, and “significant” is 0.0097, 0.0951, 0.2532, 0.3649 and 0.2772, respectively. To get the final evaluation result, linguistic terms of the risk level can be converted into crisp values using five-point Likert scale (Loh et al., 2017). Therefore, let risk grade set *V* = { very low, low, medium, high, significant } =  { 1, 2, 3, 4, 5 }. By applying the weighted average algorithm, which is the frequently used method to conduct the defuzzification of the evaluation results due to its simplicity and high efficiency (Jia et al., 2022), the fuzzy comprehensive evaluation results are converted into a crisp number 3.805. That is, the overall risk of AU_1_ falls between medium and high.

The pre-audit risk score of all auditable units ($AU_{m}$) can be obtained by repeating the above calculation. And then the risk reduction value can be estimated by applying Eqs. (2) and (3). An overview of the auditable units is given in Table 5, including the calculated pre-audit risk level and risk reduction value. The top five auditable units ranked by overall risk level are AU_23_, AU_9_, AU_27_, AU_35_, and AU_19_.

**Table 5:** Dataset by auditable unit

AU	Pre-audit risk	Small audit		Moderate audit		Large audit		Full audit		External hour	ERM	Mgt request	Hot spot	Strategic focus	AC interest	Advisory service
		*RR*_*m1*_	T_*m1*_	*RR*_*m2*_	T_*m2*_	*RR*_*m3*_	T_*m3*_	*RR*_*m4*_	T_*m4*_							
1	3.8050	1.3698	320	2.0547	480	2.7396	760	3.4245	1200	0%	No	Yes	No	No	No	No
2	3.6653	1.3195	320	1.9793	480	2.6390	760	3.2988	1200	0%	Yes	Yes	No	No	No	No
3	3.1268	1.1256	200	1.6885	320	2.2513	500	2.8141	800	0%	Yes	Yes	No	Yes	Yes	No
4	3.4528	1.2430	400	1.8645	520	2.4860	800	3.1075	1280	50%	No	Yes	No	No	Yes	No
5	2.6900	0.9684	320	1.4526	480	1.9368	760	2.4210	1200	0%	No	No	No	No	No	No
6	3.3584	1.2090	280	1.8135	400	2.4180	600	3.0226	880	0%	No	Yes	No	No	No	No
7	2.9621	1.0664	400	1.5995	520	2.1327	800	2.6659	1280	0%	No	No	No	No	No	No
8	3.4050	1.2258	200	1.8387	320	2.4516	500	3.0645	800	0%	No	No	No	No	No	No
9	4.0721	1.4660	360	2.1989	520	2.9319	800	3.6649	1200	30%	No	No	No	No	No	No
10	3.5520	1.2787	360	1.9181	520	2.5574	800	3.1968	1200	30%	No	No	No	No	No	No
11	3.4663	1.2479	400	1.8718	520	2.4957	800	3.1197	1240	30%	No	Yes	No	Yes	No	No
12	2.6958	0.9705	320	1.4557	440	1.9410	720	2.4262	1120	30%	No	No	No	No	No	No
13	2.8077	1.0108	200	1.5162	320	2.0215	500	2.5269	800	0%	No	Yes	No	No	No	No
14	2.5358	0.9129	120	1.3693	240	1.8258	440	2.2822	760	30%	No	Yes	No	No	No	No
15	3.3275	1.1979	120	1.7969	240	2.3958	440	2.9948	760	30%	Yes	No	No	No	No	No
16	3.2534	1.1712	200	1.7568	320	2.3424	480	2.9281	800	30%	No	Yes	No	No	No	No
17	3.1492	1.1337	200	1.7006	280	2.2674	400	2.8343	600	0%	Yes	Yes	No	No	Yes	No
18	3.0012	1.0804	200	1.6206	240	2.1609	360	2.7011	560	0%	Yes	Yes	No	No	Yes	No
19	4.0015	1.4405	240	2.1608	300	2.8811	400	3.6014	600	0%	Yes	Yes	No	Yes	Yes	No
20	3.2488	1.1696	240	1.7544	300	2.3391	400	2.9239	600	30%	Yes	No	No	No	No	No
21	3.2064	1.1543	200	1.7315	240	2.3086	400	2.8858	600	30%	No	No	No	No	No	No
22	3.3097	1.1915	200	1.7872	300	2.3830	480	2.9787	700	0%	Yes	Yes	No	Yes	No	No
23	4.1198	1.4831	600	2.2247	720	2.9663	1000	3.7078	1500	0%	Yes	Yes	No	Yes	Yes	Yes
24	3.0305	1.0910	280	1.6365	360	2.1820	520	2.7275	720	100%	No	Yes	No	Yes	No	Yes
25	2.5607	0.9219	360	1.3828	420	1.8437	600	2.3046	800	100%	Yes	Yes	No	No	Yes	No
26	3.4342	1.2363	400	1.8545	500	2.4726	640	3.0908	800	30%	Yes	Yes	No	Yes	No	Yes
27	4.0549	1.4598	360	2.1896	440	2.9195	600	3.6494	800	100%	Yes	Yes	Yes	No	Yes	No
28	2.7853	1.0027	80	1.5041	160	2.0054	400	2.5068	700	0%	No	Yes	No	No	No	Yes
29	3.0266	1.0896	160	1.6344	240	2.1792	400	2.7239	600	0%	No	No	Yes	No	No	No
30	3.3786	1.2163	400	1.8244	500	2.4326	640	3.0407	800	0%	No	Yes	No	No	No	No
31	3.2497	1.1699	240	1.7548	400	2.3398	600	2.9247	1000	0%	Yes	Yes	No	No	No	No
32	3.2020	1.1527	320	1.7291	400	2.3054	520	2.8818	660	0%	Yes	Yes	Yes	No	No	Yes
33	3.3896	1.2203	280	1.8304	400	2.4405	600	3.0506	880	0%	No	Yes	Yes	Yes	No	No
34	2.6792	0.9645	80	1.4468	120	1.9290	200	2.4113	400	0%	No	Yes	No	No	Yes	Yes
35	4.0209	1.4475	100	2.1713	200	2.8950	400	3.6188	720	0%	Yes	Yes	No	Yes	Yes	Yes
36	3.4882	1.2558	680	1.8836	880	2.5115	1160	3.1394	1600	0%	No	Yes	Yes	Yes	Yes	No
37	3.4142	1.2291	300	1.8437	400	2.4582	600	3.0728	900	0%	No	No	No	No	No	No
38	3.3006	1.1882	160	1.7823	280	2.3764	520	2.9705	880	0%	No	No	No	Yes	Yes	No
39	3.2391	1.1661	400	1.7491	520	2.3322	720	2.9152	960	30%	Yes	No	No	Yes	No	No
40	3.5333	1.2720	200	1.9080	320	2.5440	560	3.1800	920	0%	Yes	No	Yes	Yes	Yes	Yes
41	3.4904	1.2565	120	1.8848	200	2.5131	300	3.1414	500	0%	No	Yes	No	No	No	No
42	3.1093	1.1193	300	1.6790	400	2.2387	520	2.7984	700	0%	Yes	No	No	Yes	Yes	No
43	3.7986	1.3675	360	2.0512	500	2.7350	700	3.4187	1000	0%	Yes	Yes	Yes	No	Yes	No
44	3.3049	1.1898	320	1.7846	480	2.3795	700	2.9744	1000	0%	No	Yes	No	No	No	No
45	2.8118	1.0122	400	1.5184	600	2.0245	900	2.5306	1300	0%	No	No	Yes	No	Yes	No

Other information (*e.g.*, working hours and nature of the audit) needed for making decision are provided by the senior audit manager. For instance, performing a small-scope audit at AU_1_ could take 320 h and reduce existing risk by 1.3698. Similarly, conducting a moderate (large or full) audit requires 480 (760 or 1200) hours and can reduce the risk by 2.0574 (2.7396 or 3.425). Working hours of audit managers who are responsible for supervising the audit are excluded from the estimated audit time. This audit is not an advisory service and is assigned to in-house audit team only (*i.e.*, external consultant accounts for 0% of the total work time). Management used to request audit team to conduct this audit. However, this audit topic is not related to enterprise risk management (ERM), does not belong to the industry hot spot, is not corporate strategic focus, nor within the potential scope of audit committee (AC)’s interest. On the other hand, considering the restriction of international travel due to COVID-19 pandemic, external consultants can provide local audit support of overseas entities such as AU_9_, which could account for 30% of total work time. In other words, the in-house audit team would concurrently complete the rest 70% of audit tasks remotely using technology. IT audit (*e.g.*, AU_27_) will be fully (100%) completed by consultants due to lack of proficient IT auditors in the company.

#### Audit area selection

The available audit resources considered during the annual audit planning include: (1) A total of 9,600 working hours of 6 internal auditors who execute the audit plan. (2) The approved 2022 annual budget for hiring external consultants is RMB 2 million (~ USD 312,500). With this fund, the IAF can hire external resources up to 1,950 h based on consultant’s hourly rate. Therefore, 11,550 h are available for carrying out the audit plan.

According to the characteristics of the integrated risk-based audit plan and the practice of the studied IAF, the goals of the case company are expressed as follows. (1) reduce risk level as much as possible; (2) the more audit areas linked with ERM the better; (3) accommodate as many management requests as possible; (4) cover industry audit hot spots as many as possible; (5) cover company’s strategy as many as possible; (6) consider potential interest of audit committee/board and the more the better; and (7) spend as much time as possible on advisory service. Table 6 provides a summary of goal weights, aspirations, penalty weights for below/above each goal given by the senior manager. The importance of each goal also can be generated by MCDA method such as AHP.

**Table 6:** Goal description

Goals	Weights	Lower bound	Upper bound	Penalty weights for below the goal	Penalty weights for above the goal
G1: Risk reduction	0.2	38	66	5	
G2: Linkage with ERM	0.2	5,775	11,550	5	
G3: Management request	0.15	5,775	11,550	3	
G4: Audit hot spots	0.07	600	7,760	2	
G5: Strategic focus	0.12	2,310	11,550	2	
G6: Interest of audit committee	0.16	3,465	11,550	3	
G7: Advisory service	0.1	1,155	3,465	2	3

Each goal is explained as follows based on the above dataset.

(G1): Existing risks should at least be reduced by 38, the more the better. Risk reduction is the core of the integrated risk-based audit planning. To avoid getting the result value less than the lower bound and make the result value the higher the better, the senior audit manager assigns the penalty weight of 5 for below the goal.
(G2): Audit time spent on areas related to ERM must be over 5775 h (or 50% of total available time, which is also the upper bound), the more the better. Addressing ERM is an essential way for the IAF to target the pulse of the company, thereby a penalty weight of 5 is assigned to this goal.
(G3): Audit time spent on areas proposed by management must be over 5775 h (or 50% of total available time), the more the better. Management concerns are a key indicator of business needs and should be considered in the annual audit planning process. Hence, a penalty weight of 3 is assigned for below the goal.
(G4): Audit time spent on hot audit topics in the industry must be over 600 h (or about 5% of total available time), the more the better. Covering hot spots helps to keep an eye on the industrial trend. Hence, a penalty weight of 2 is assigned for below the goal.
(G5): Audit time spent on areas related to company’s strategy must be over 2310 h (or 20% of total available time), the more the better. A penalty weight of 3 is assigned for below the goal.
(G6): At least 3465 h (or 30% of total available time) are spent on areas which would be the interest of audit committee, the more the better. Audit committee’s opinion is an important input and thus a penalty weight of 3 is assigned for below this goal.
(G7): At least 1155 h (or 10% of total available time) are spent on advisory service, the more the better. Senior audit manager assigns penalty weight of 2 for below the advisory service goal. However, as assurance service is still the main task of the IAF, 3465 h (or 30% of total available time) are set as the upper bound. Also, to avoid too many hours spent on the advisory service, a penalty weight of 3 is assigned for exceeding the goal.

Formulation of the audit planning problem is expressed as follows.

12 $$ \begin{gathered} {\text{Minimize}}{\kern 1pt} {\kern 1pt} {\kern 1pt} {\kern 1pt} 0.2 \times (d_{1}^{ + } + 5d_{1}^{ - } + e_{1}^{ + } + e_{1}^{ - } ){ + }0.2 \times (d_{2}^{ + } + 5d_{2}^{ - } + e_{2}^{ + } + e_{2}^{ - } ){ + }0.15\hfill\\ \times (d_{3}^{ + } + 3d_{3}^{ - } + e_{3}^{ + } + e_{3}^{ - } ) \hfill \\ { + }0.07 \times (d_{4}^{ + } + 2d_{4}^{ - } + e_{4}^{ + } + e_{4}^{ - } ){ + 0}{\text{.12}} \times (d_{5}^{ + } + 2d_{5}^{ - } + e_{5}^{ + } + e_{5}^{ - } ){ + }0.16\hfill\\ \times (d_{6}^{ + } + 3d_{6}^{ - } + e_{6}^{ + } + e_{6}^{ - } ) \hfill \\ { + }0.1 \times (3d_{7}^{ + } + 2d_{7}^{ - } + e_{7}^{ + } + e_{7}^{ - } ) \hfill \\ \end{gathered} $$

13 $$ \begin{gathered} {\text{s}}.{\text{t}}. \hfill \\ \sum\limits_{m = 1}^{45} {\sum\limits_{n = 1}^{4} {RR_{mn} \times X_{mn} } } - d_{1}^{ + } + d_{1}^{ - } = y_{1} ,\,\,\,\,m = 1,2,...,45;\,\,\,\,n = 1,2,3,4 \hfill \\ \end{gathered} $$

14 $$ y_{1} - e_{1}^{ + } + e_{1}^{ - } = 66 $$

15 $$ {\kern 1pt} 38 \le y_{1} \le 66 $$

16 $$ \sum\limits_{m = 1}^{45} {\sum\limits_{n = 1}^{4} {T_{{mn({\text{erm}})}} } } \times X_{mn} - d_{2}^{ + } + d_{2}^{ - } = y_{2} ,\,\,\,\,m = 1,2,...,45;\,\,\,\,n = 1,2,3,4 $$

17 $$ y_{2} - e_{2}^{ + } + e_{2}^{ - } = 11,550 $$

18 $$ {\kern 1pt} 5,775 \le y_{2} \le 11,550 $$

19 $$ \sum\limits_{m = 1}^{45} {\sum\limits_{n = 1}^{4} {T_{{mn({\text{mgt}})}} } } \times X_{mn} - d_{3}^{ + } + d_{3}^{ - } = y_{3} ,\,\,\,\,m = 1,2,...,45;\,\,\,\,n = 1,2,3,4 $$

20 $$ y_{3} - e_{3}^{ + } + e_{3}^{ - } = 11,550 $$

21 $$ {\kern 1pt} 5,775 \le y_{3} \le 11,550 $$

22 $$ \sum\limits_{m = 1}^{45} {\sum\limits_{n = 1}^{4} {T_{{mn({\text{hot}})}} } } \times X_{mn} - d_{4}^{ + } + d_{4}^{ - } = y_{4} ,\,\,\,\,m = 1,2,...,45;\,\,\,\,n = 1,2,3,4 $$

23 $$ y_{4} - e_{4}^{ + } + e_{4}^{ - } = 7,760 $$

24 $$ {\kern 1pt} 600 \le y_{4} \le 7,760 $$

25 $$ \sum\limits_{m = 1}^{45} {\sum\limits_{n = 1}^{4} {T_{{mn({\text{stgy}})}} } } \times X_{mn} - d_{5}^{ + } + d_{5}^{ - } = y_{5} ,\,\,\,\,m = 1,2,...,45;\,\,\,\,n = 1,2,3,4 $$

26 $$ y_{5} - e_{5}^{ + } + e_{5}^{ - } = 11,550 $$

27 $$ {\kern 1pt} 2,310 \le y_{5} \le 11,550 $$

28 $$ \sum\limits_{m = 1}^{45} {\sum\limits_{n = 1}^{4} {T_{{mn({\text{ac}})}} } } \times X_{mn} - d_{6}^{ + } + d_{6}^{ - } = y_{6} ,\,\,\,\,m = 1,2,...,45;\,\,\,\,n = 1,2,3,4 $$

29 $$ y_{6} - e_{6}^{ + } + e_{6}^{ - } = 11,550 $$

30 $$ {\kern 1pt} 3,465 \le y_{6} \le 11,550 $$

31 $$ \sum\limits_{m = 1}^{45} {\sum\limits_{n = 1}^{4} {T_{{mn({\text{adv}})}} } } \times X_{mn} - d_{7}^{ + } + d_{7}^{ - } = y_{7} ,\,\,\,\,m = 1,2,...,45;\,\,\,\,n = 1,2,3,4 $$

32 $$ y_{7} - e_{7}^{ + } + e_{7}^{ - } = 3,465 $$

33 $$ {\kern 1pt} 1,155 \le y_{7} \le 3,465 $$

34 $$ d_{k}^{ + } ,{\kern 1pt} {\kern 1pt} {\kern 1pt} d_{k}^{ - } ,{\kern 1pt} {\kern 1pt} e_{k}^{ + } ,{\kern 1pt} e_{k}^{ - } \ge 0,\;\,\,k = 1,2,...,7 $$

35 $$ \sum\limits_{m = 1}^{45} {\sum\limits_{n = 1}^{4} {T_{{mn({\text{int}} )}} } } \times X_{mn} \le 9,600,\,\,\,\,m = 1,2,...,45;\,\,\,\,n = 1,2,3,4 $$

36 $$ \sum\limits_{m = 1}^{45} {\sum\limits_{n = 1}^{4} {T_{{mn({\text{ext}})}} } } \times X_{mn} \le 1,950,\,\,\,\,m = 1,2,...,45;\,\,\,\,n = 1,2,3,4 $$

37 $$ \sum\limits_{n = 1}^{4} {X_{mn} \le 1} ,\;\;\;\;m = 1,2,...,45 $$

38 $$ X_{mn} = 0\;{\text{or}}\;1,\,\,\,\,m = 1,2,...,45;\,\,\,\,n = 1,2,3,4 $$

39 $$ {12} \le \sum\limits_{m = 1}^{45} {\sum\limits_{n = 1}^{4} {X_{mn} } } \le {18} $$

40 $$ \sum\limits_{n = 1}^{4} {X_{4n} = 1} ,\,\,\,\,\sum\limits_{n = 1}^{4} {X_{17n} = 1} ,\,\,\,\,\sum\limits_{n = 1}^{4} {X_{18n} = 1} $$

where Eqs. (13) to (34) are the seven goals and the deviations between the realized results and the desired results. $T_{{mn({\text{erm}})}}$, $T_{{mn({\text{mgt}})}}$, $T_{{mn({\text{hot}})}}$, $T_{{mn({\text{stgy}})}}$, $T_{{mn({\text{ac}})}}$, $T_{{mn({\text{adv}})}}$ are respectively the amount of time under $n$ th audit scope for $m$ th auditable unit related to ERM, management request, industry audit hot spot, organizational strategy focus, interest of audit committee/board, and advisory service. Equation (35) and (36) ensure that the audit hours spent by in-house internal auditors and external consultants are within the budgeted hours. Equation (37) means that an audit only can be conducted under one work scope. In Eq. (38), $X_{mn}$ is the binary variable to decide whether to select $m$ th auditable unit at $n$ th work scope. Equation (39) indicates that the total number of audits to be performed in a year is between 12 and 18. As repetitive audits (*e.g.*, continuous audits of employee expense report and vendor payment, and mandatory internal audit by local regulation) must be selected, Eq. (40) identifies three audits that must be included in the audit plan.

### Results discussion

**Fig. 2:**
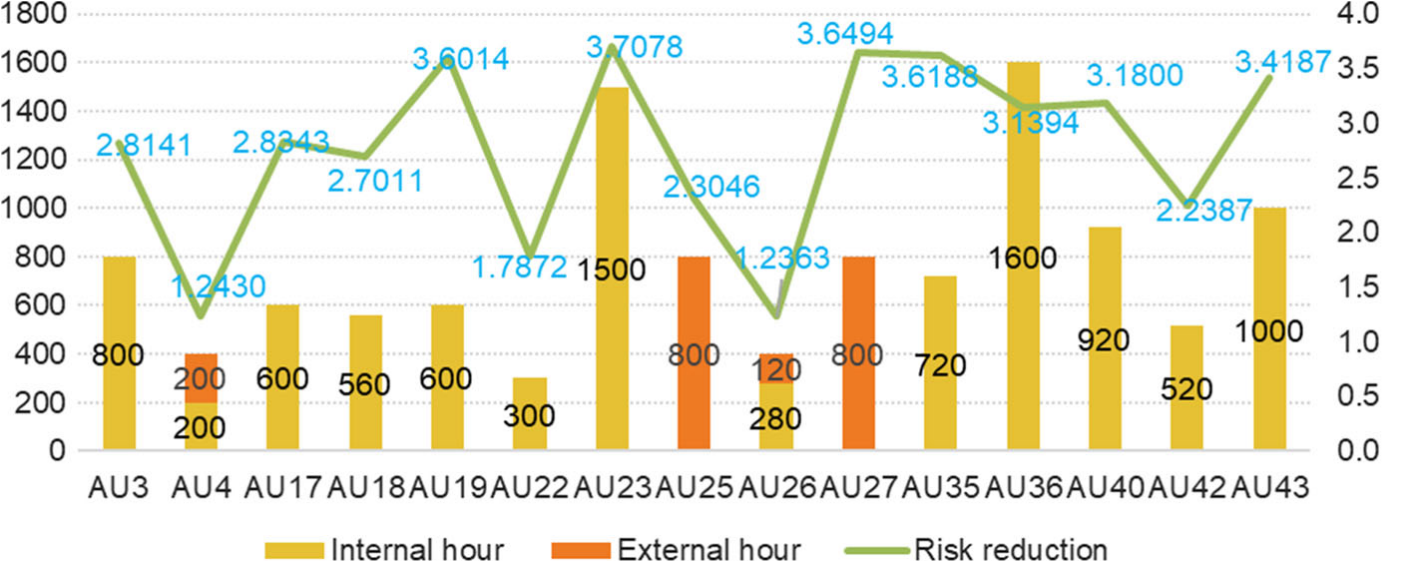
An overview of the results

Using the dataset in Table 5, the problem is solved with LINGO 17.0 software (Lindo Systems, Chicago, IL, USA). Figure 2 provides the final results of the selected auditable units, audit time and risk reduction value.

As it is seen, 15 out of 45 auditable units are selected for the audit, including 4 advisory engagements (*AU*_*23*_, *AU*_*26*_, *AU*_*35*_ and *AU*_*40*_) and the other 11 assurance engagements. Among the selected audits, small audits are performed at 2 auditable units (*AU*_*4*_ and *AU*_*26*_), a moderate audit is undertaken at 1 auditable unit (*AU*_*22*_) and a review of large scope is performed at 1 auditable unit (*AU*_*42*_), and the rest 11 auditable units (*AU*_*3*_, *AU*_*17*_, *AU*_*18*_, *AU*_*19*_, *AU*_*23*_, *AU*_*25*_, *AU*_*27*_* AU*_*35*_, *AU*_*36*_, *AU*_*40*_ and *AU*_*43*_) are subject to full audits. This result is aligned with the methodology of the studied IAF that intends to conduct deeper audits to dig out more values for the business. The developed audit plan would take 11,520 h. All the available time of internal team (9600 h) are fully utilized, and 98.5% of external consultant time (1920 h) will be used.

**Fig. 3:**
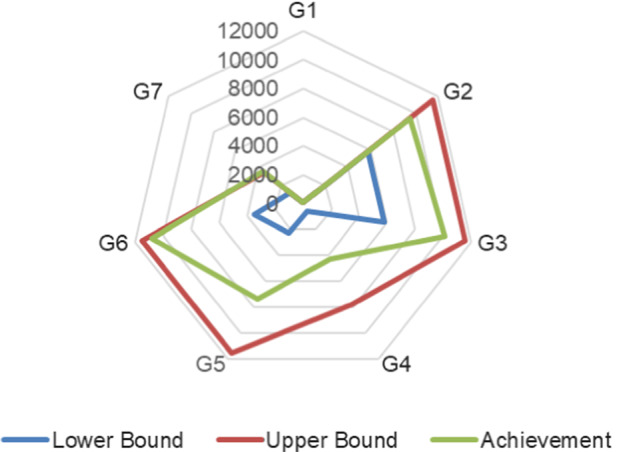
Comparison between achieved result and target

In addition, Fig. 3 depicts the realized goals. All the goals can be achieved as follows according to the optimal solution.

(G1): A total risk reduction of 41.5 is achieved, which is 9.14% higher than the desired risk reduction value.
(G2): It would take 9520 h to complete 13 audit engagements relevant with ERM, which is 65% higher than the expectation.
(G3): It would take 10,080 h to perform 13 engagements related to management request, which is 75% higher than the expectation.
(G4): There are 4320 h spent on 4 audit engagements covering industry hot spots. The achieved result is six times higher than the expectation.
(G5): There are 7360 h spent on 9 audit engagements covering company’s strategy. The achieved result is more than three times as many as the desired hours.
(G6): There are 10,820 h spent on 13 engagements addressing the interest of audit committee, which is twice higher than the expectation.
(G7): There are 3540 h spent on 4 advisory engagements, which is slightly (75 h) above the upper bound and is three times as many as the pre-defined requirement.

### Sensitivity analysis

To check the robustness of the solution, two sensitivity analyses are presented regarding changes in DMs’ weights on the risk items and goals.

**Fig. 4:**
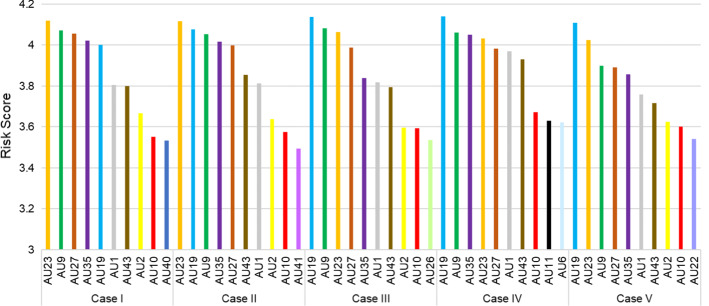
Top 10 risky areas by different weights

First, when assessing the overall risk of auditable units using FCE method, the illustrative case, namely Case I, is based on one set of weights $W$ = (0.2716, 0.2256, 0.3509, 0.1519) from AHP method. Specifically, DMs view operational risk as the most important risk to the organization, followed by strategic risk, financial risk, and compliance risk. To make strategic risk the most critical one, let Case II exchange the weight of strategic risk for that of operational risk in the original weight vector, thereby $W$ = (0.3509, 0.2256, 0.2716, 0.1519). Case III makes a weight swap between financial risk and operational risk in the original weight vector, and $W$ = (0.2716, 0.3509, 0.2256, 0.1519). Similarly, compliance risk becomes the most important one in Case IV, and $W$ = (0.2716, 0.2256, 0.1519, 0.3509). In addition, Case V makes the weight of each main criterion and sub criterion equal, that is, $W$ = [1]_4*4_, $w_{1}$ = [1]_6*6_, $w_{2}$ = [1]_3*3_, $w_{3}$ = [1]_6*6_, $w_{4}$ = [1]_3*3_. Figure 4 depicts the comparison of final evaluation results by top 10 risky auditable units.

As it is seen, *AU*_23_ has the highest risk score in both Case I and Case II but not in other cases. Therefore, this auditable unit is not sensitive to strategic risk but is sensitive to financial and compliance risks. Also, *AU*_19_ ranks fifth in the illustrative case, but ranks either the first or the second in other cases. Therefore, it is sensitive to other risks. Besides, *AU*_23_, *AU*_9_, *AU*_27_, *AU*_35_, *AU*_19_, *AU*_1_, and *AU*_43_ are the top 7 risky auditable units in all cases, while the specific rankings vary among the cases. In general, these auditable units are viewed as high-risk areas and the impact of weight changes on the ranking is not obvious. On the other hand, *AU*_40_ becomes top 10 risky area only in Case I, and *AU*_2_ is not ranked as top 10 risky area only in Case IV. To summarize, DMs’ judgements on the importance of risks could impact the risk evaluation results to a certain extent. However, the difference of the risk score of each auditable unit is not significant among the cases, ranging from 0.062 to 0.4321, or from 2 to 14%.

**Fig. 5:**
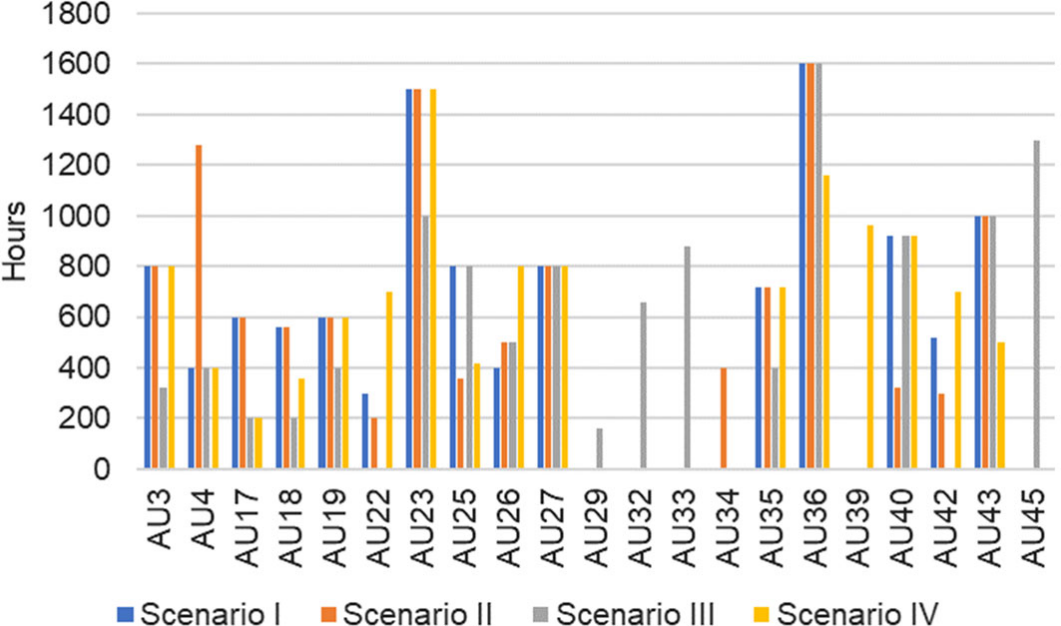
Comparison of selection results

Second, as given in Table 7, this study sets different weights of goals in determining the multi-objective selection of auditable units. The illustrated case study, Scenario I, emphasizes on risks. The assumed other three scenarios emphasize other objectives. With all constraints unchanged, the solutions are presented in Fig. 5.

**Table 7:** Weights of different selection strategies

Goals	Weights of the goals			
	Scenario I	Scenario II	Scenario III	Scenario IV
G1: Risk reduction	0.2	0.1	0.1	0.1
G2: Linkage with ERM	0.2	0.1	0.1	0.3
G3: Management request	0.15	0.3	0.05	0.1
G4: Audit hot spots	0.07	0.05	0.3	0.05
G5: Strategic focus	0.12	0.1	0.1	0.3
G6: Interest of audit committee	0.16	0.3	0.05	0.1
G7: Advisory service	0.1	0.05	0.3	0.05
Solution	15 AUs	16 AUs	17 AUs	16 AUs
Utilized audit time (hours)	11,520	11,540	11,540	11,540

Scenario I considers risk reduction (G1) and linkage with ERM (G2) as key goals while balancing other goals. As a result, 15 audits are selected. Scenario II cares more about stakeholders. Under this strategy, management request (G3) and AC’s interest (G6) are given higher weights. Finally, 16 audits are selected. In Scenario III, the nature of the audit is emphasized and thus hot topics (G4) and advisory service (G7) are assigned with higher weights, and it ends up with 17 selected audits. Scenario IV views enterprise risk management (G2) and company strategy (G5) as critical factors. As a result, 16 audits are selected. The available audit time are almost fully utilized in all scenarios. As illustrated, there are 13 audits in common under all scenarios although the work scope varies among the scenarios. For example, compared with Scenario I, Scenario II selects one additional audit *AU*_*27*_. To compensate the time spent on this extra audit, the audit scope/effort is different for 6 out of the 15 audits in common.

### Management feedback on the framework

The proposed framework for developing integrated risk-based audit plan enhances the value of audit work that brings to the organization.

Because the case study used management judgement and data that were not considered in the manually performed planning process, a direct comparison between the proposed solution and the real-life audit plan was not feasible. However, the designed process is reasonable and the results are satisfactory to audit leaders. The new framework not only selects more critical audits, but also determines the effort level to reduce low value work.

According to the senior audit manager, *“Unlike the look back approach we are using now, the proposal adopts a proactive approach. This is the mindset we need to implement to show how people can make decision. The proposed framework makes much more sense in the data driven audit planning, and we will need it when it becomes hard for us to reduce potential audits and pick more meaningful projects at one point. Although we are not yet data driven audit team because the current management team of the company is not at this appetite, this could change next day”*. In addition, as per the CAE, *“The framework is a good way to track and explain the selection decision. You also bring up a good point to start resource plan upfront selection. How we operate now is that we work backward to manage the numbers at the last minute. However, it is not necessary to use this new approach at this moment because we do not have a large population of auditable unit to make the selection now. Our candidate topics are mainly from management input meetings. We pretty much know what we are going to select as we kind of understand which areas would executives like us to check. With that said, I’m not denying the benefits of the approach and the promising results. This is the way to future. This will be useful as we grow up and become more complex considering the ongoing and future mergers and acquisitions. I’m also thinking of applying the approach to individual audit engagement, we can figure out where else we can apply this approach best”.*

## Conclusion and future work

Internal audit planning is a methodical process of selection and resource allocation. This paper presents a novel decision support model for annual planning problem based on OR methods. The proposed multi-stage framework synthesizes DMs’ judgements on risk rating and considers various goals and constraints in selecting audit engagements and allocating resources.

Starting from the preparation of the audit and risk universe, the proposed framework uses a combined AHP and FCE method for assessing the existing risk of each auditable unit. This method aggregates DMs’ opinions about organizational risks. Then the risk reduction value is estimated based on the obtained risk level and possible audit scopes. Finally, WMCGP model is adopted to select candidate audits and allocate resources to the audit engagement concurrently, aiming to achieve multiple objectives. The proposed integrated risk-based audit planning enables the IAF to consider value-added factors in addition to risk management. A real-life case of a manufacturing company is presented to elaborate how the proposed framework can be applied. However, the proposed framework can be applied to any organization.

This study has some theoretical implications for auditing in general and risk-based audit planning in particular. It extends existing internal auditing research which is an important but under-researched area. The new risk model of the manufacturing industry, and the exploration of the relationship between risk mitigation and audit time also contribute to the body of knowledge on risk management. Moreover, this interdisciplinary study sheds new insights into the audit planning process. Compared with previous studies which use ranking methods for audit project selection and only focus on a single goal of risk mitigation, the proposed framework enables simultaneous consideration of resource allocation and project selection for achieving multiple objectives. In addition to the theoretical implications, the findings of this study support the IAF in developing a value-added annual plan according to the departmental strategy. With the proposed framework, internal audit planning can be conducted in a justified, scientific, and transparent way, which enhances the reliability of internal audit activities. This study also provides a reference for audit software developers to improve the design of audit planning module. In fact, the implications of this paper are not limited to annual audit planning problem, the proposed model would be of great practical value for many decision-making problems, especially for selection, allocation, and evaluation in various scenarios.

In terms of the study limitation, an issue may arise is the increasing amount of effort for audit planning work. When the size of the audit universe is small, managers can easily make decisions based on their experience. Moreover, a decision analyst might be needed to implement the proposed framework. However, with repeated use of the model, the effort level can be reduced. Also, for an organization to embrace integrated risk-based audit planning, the risk management system should be sufficiently mature. Future studies could apply the proposed framework to various organizations in different countries and engage more practitioners in the data collection. In AHP-group decision making, other aggregation methods, such as Bayesian approach and Delphi technique, can be employed to achieve a consensus for future research. In determining the membership degree using FCE method, other membership functions, such as triangular or trapezoidal function and even nonlinear functions can be used. More studies on risk reduction value would also be beneficial to auditing research.

## Data Availability

The data that supports the findings of this study are partially included in this article. The complete data are available from the corresponding author upon request.
